# Ameliorative Effects of *Acacia* Honey against Sodium Arsenite-Induced Oxidative Stress in Some Viscera of Male Wistar Albino Rats

**DOI:** 10.1155/2013/502438

**Published:** 2013-12-03

**Authors:** Muhammad Aliyu, Sani Ibrahim, Hajiya M. Inuwa, Abdullahi B. Sallau, Olagunju Abbas, Idowu A. Aimola, Nathan Habila, Ndidi S. Uche

**Affiliations:** Department of Biochemistry, Ahmadu Bello University, Zaria 810271, Kaduna State, Nigeria

## Abstract

Cancer is a leading cause of death worldwide and its development is frequently associated with oxidative stress-induced by carcinogens such as arsenicals. Most foods are basically health-promoting or disease-preventing and a typical example of such type is honey. This study was undertaken to investigate the ameliorative effects of *Acacia* honey on sodium arsenite-induced oxidative stress in the heart, lung and kidney tissues of male Wistar rats. Male Wistar albino rats divided into four groups of five rats each were administered distilled water, *Acacia* honey (20%), sodium arsenite (5 mg/kg body weight), *Acacia* honey, and sodium arsenite daily for one week. They were sacrificed anesthetically using 60 mg/kg sodium pentothal. The tissues were used for the assessment of glutathione peroxidase, catalase, and superoxide dismutase activities, protein content and lipid peroxidation. Sodium arsenite significantly (*P* < 0.05) suppressed the glutathione peroxidase, catalase, superoxide dismutase activities with simultaneous induction of lipid peroxidation. Administration of *Acacia* honey significantly increased (*P* < 0.05) glutathione peroxidase, catalase, and superoxide dismutase activities with concomitant suppression of lipid peroxidation as evident by the decrease in malondialdehyde level. From the results obtained, *Acacia* honey mitigates sodium arsenite induced-oxidative stress in male Wistar albino rats, which suggest that it may attenuate oxidative stress implicated in chemical carcinogenesis.

## 1. Introduction

Honey is a viscous, supersaturated sugar solution derived from nectar gathered and modified by the honeybee, *Apis mellifera *[1]. It is composed of at least 300 compounds having fructose and glucose in highest concentrations. A great variety of minor components, including phenolics and flavonoids, different enzymes, carotenoids, organic acids, and proteins are also present [1–4]. *Acacia* honey is a type of honey produced by bees in the *Acacia* flowers, hence the name. Earlier report from our laboratory demonstrated that daily administrations of *Acacia* honey to Wistar rats have both positive and negative biological effects [5]. We have also demonstrated its antiproliferative effects against prostate cancer cell line [6] and lung cancer cell line *in vitro* [7]. Contrariwise, we have demonstrated that fractionation of *Acacia* honey negatively affected its antioxidant potentials by making it a radical generating agent in contrast with the unfractionated sample. Indeed, the antioxidant potential of the whole *Acacia* honey was comparable with *α*-tocopherol; a well-known standard antioxidant [8]. Characterization of *Acacia* honey revealed three (3) phenolic acids [p-hydroxybenzoic, ferulic, and t-cinnamic acid], five (5) free flavonoids [pinobanksin, apigenin, pinocembrin, chrysin, and acacetin] and abscisic acid [9].

Free radicals cause oxidative damage to lipids, proteins and nucleic acids leading to many biological complications including carcinogenesis, mutagenesis, aging, and atherosclerosis[10]. Arsenic has been implicated in covalent interactions with the thiol groups in proteins causing instability on the structure-function relationship of proteins. Arsenic compounds, which are ubiquitous in nature, are released into the environment via industrial or agricultural processes as well as some medical applications [11]. Consumption of arsenicals through contaminated water is prevalent in many areas of the world [12]. Arsenical applications in insecticides, acaricides, and soaps are a major risk factor to individuals who are exposed to them. Sodium arsenite is an agent that causes chromosomal breakage [13], which can interact with other substances like metals, thereby potentiating its effects [14]. Its administration has been reported to compromises the integrity of the liver, of mouse, rat, fish, and goat [15–17]. Although studies have been conducted on the *Acacia* honey in relation to oxidative stress in brain, liver and plasma, to the best of our knowledge no such work has been done on heart, lung and kidney tissues with sodium arsenite in the presence of *Acacia* honey. Therefore, this study was undertaken to investigate the effects of *Acacia* honey on sodium arsenite-induced oxidative stress in the heart, lung, and kidney tissues of male Wistar rats. This work will further contribute to the existing knowledge in the area of functional foods-based research.

## 2. Materials and Methods

### 2.1. Chemicals and Reagents

Sodium arsenite (5 mg/kg body weight) equivalent to two-tenth of the oral LD_50_ was used [18] in all experiment. *Acacia* honey was dissolved in distilled water to prepare a 20% (v/v) honey solution and 5 mL/kg body weight was used [5]. All other reagents and chemicals used were of analytical grade.

### 2.2. Sample Collection

The *Acacia* honey was collected from Achida town, Wurno Local Government Area, Sokoto State, Nigeria, in December 2012, from *Acacia *flower. It was identified by pollen grain analysis and maintained at the Department of Biochemistry, Ahmadu Bello University, Zaria, Nigeria. It was kept at 4°C until analysis.

### 2.3. Experimental Animals and Design

Twenty (20) male albino rats of Wistar (150–195 g) were used in the present study. They were allowed to adapt for one week under standard laboratory conditions of 12-hour light-dark cycle before commencement of all experiments. The rats were maintained in the animal house of Department of Biochemistry, Ahmadu Bello University, Zaria, Nigeria with protocol for the study approved by the Institutional Animal right review committee. During acclimatization, the rats were allowed free access to NIH-07 pelletized diet and water. In addition, all rats were humanely cared for in accordance with the National Institute of Health (NIH) Guide for the care and use of Laboratory Animals. They were randomly divided into four (4) groups of five (5) rats each. The animals were treated daily for one week as shown in the experimental design as follows.

 Group 1: distilled water (normal control).
 Group 2: 20% v/v *Acacia* honey at 5 mL/kg body weight.
 Group 3: 5 mg/kg body weight sodium arsenite (positive control).
 Group 4: 20% v/v *Acacia* honey + 5 mg/kg body weight sodium arsenite.

### 2.4. Collection and Preparation of Samples

Twenty four (24) hours after the administration of the last treatment, the rats were kept without food overnight and were humanely sacrificed with sodium pentothal (60 mg/kg body weight). Heart, lung, and kidney tissues were collected. The tissues were homogenized in 1 : 5 of 0.9% sodium chloride (ice-cold). The supernatant was collected after centrifugation at 3500 ×g at 4°C for 10 minutes and kept at −80°C until further analysis. Each time the supernatant was outside the freezer, it was kept in ice bags. By using Auto Analyzer Hitachi Roche 7020 (902, Japan Inc.), the total protein contents of the tissues were determined based on the standard manufacturer's protocol.

### 2.5. Determination of Oxidative Stress Biomarkers

Lipid peroxidation was determined by measuring malondialdehyde (MDA) formed by thiobarbituric acid reaction [19]. Catalase (CAT) activity was estimated by measuring the rate of decomposition of H_2_O_2_ [20]. The level of superoxide dismutase (SOD) activity was determined by the method of Misra and Fridovich [21], while the method of Wendel [22] was adopted in estimating the activity of glutathione peroxidase.

### 2.6. Statistical Analysis

To address the biological variability and stability of the samples, each and every experiment was repeated at least three times and the results were expressed as mean ± standard deviation. Differences between the groups were analyzed by one-way analysis of variance (ANOVA) with the aid of Statistical Package for Social Sciences (SPSS) software, SPSS Inc., Chicago, IL, USA, Standard version 19. *P* values <0.05 were considered statistically significant for differences in mean using the least significant difference (LSD).

## 3. Results

Assessment of the oxidative stress biomarkers in the heart of the animals is presented in Table 1. The result showed that the group induced with stress using sodium arsenite but treated with 20% (v/v) honey had significantly (*P* < 0.05) reduced level of oxidative stress as evident by the lower level of MDA compared to the group induced with sodium arsenite without treatment. On the other hand, administration of 20% (v/v) honey significantly (*P* < 0.05) increased SOD, catalase, and glutathione peroxidase levels when compared to the sodium arsenite induced group without treatment. There is, however, no significant (*P* > 0.05) difference in the total protein in all the groups.

Table 2 shows the assessment of the oxidative stress biomarkers in the lungs of the animals. Here, the group induced with sodium arsenite but treated with 20% (v/v) showed honey significantly (*P* < 0.05) reduced oxidative stress as evident by the lower level of MDA compared to the group induced with sodium arsenite without treatment. Contrariwise, administration of honey (20%, v/v) significantly (*P* < 0.05) increased SOD, catalase, and glutathione peroxidase levels when compared to the sodium arsenite induced group without treatment. Again, there is no significant (*P* < 0.05) difference in the total protein in all the groups.

Table 3 depicts the assessment of the oxidative stress biomarkers in the kidney of the animals. It showed that the group induced with sodium arsenite but treated with 20% (v/v) honey significantly (*P* < 0.05) showed reduced oxidative stress as evident by the lower level of MDA compared to the group induced with sodium arsenite without treatment. On the other hand, 20% honey significantly (*P* < 0.05) increased SOD, catalase, and glutathione peroxidase levels when compared to the sodium arsenite induced group without treatment. However, the group administered with honey alone does not have significantly (*P* > 0.05) higher SOD compared to sodium arsenite induced group without treatment. There is still no significant (*P* > 0.05) difference in the total protein in all the groups.

## 4. Discussion

Antioxidants role in the maintenance of health and chemoprevention of disorders and diseases has received great attention [23]. As a result of the participation of oxidative processes in the onset and development of degenerative diseases, much attention has been paid to the antioxidant properties of foods rich in polyphenols [24]. Redox sensitive biomarkers could be seen as those enzymatic and nonenzymatic antioxidants/nonantioxidant (like MDA) that are stimulated either at cellular or molecular level in response to oxidation-reduction reactions during metabolism. Honey is a novel antioxidant because of the presence of flavonoids and phenolics as part of its constituents. In the present study, we report the assessment of redox sensitive biomarkers due to *Acacia* honey and sodium arsenite administration *in vivo*.

It has been established that free radicals cause oxidative damage to lipids, proteins, and nucleic acids leading to many biological complications including carcinogenesis, mutagenesis, aging, and atherosclerosis [10]. Arsenite interacts with thiol-containing amino acids, peptides, and proteins [25] and exerts cellular toxicity by binding to sulfhydryl groups which results in enzyme inhibition. During arsenic metabolism, oxygen radical may be produced, possibly leading to damage to DNA, proteins, lipids, and other molecules. There is a positive correlation between lipid peroxidation and arsenic tissue concentrations in the livers, kidneys, and heart of arsenite-treated rats [26]. Sodium arsenite-induced ROS, such as superoxide anions and hydroxyl radicals, exert effects directly or indirectly on cellular material [27]. It has also been reported that arsenic induces oxidative stress by multiple mechanisms [28]. Chronic arsenic exposure through drinking water to humans leads to carcinogenesis of almost all organs in the human system [29, 30]. Our results clearly show that sodium arsenite administration stimulated lipid peroxidation with simultaneous negative effects on the enzymatic antioxidants which was ameliorated by administration of *Acacia* honey. The antioxidative potentialities of honey have been reported [31, 32] and are generally attributed to its phenolic compounds and flavonoids [33–36]. Basically, various polyphenols reported in honey which include caffeic acid, caffeic acid phenyl ester, chrysin, galangin, quercetin, acacetin, kaempferol, pinocembrin, pinobanksin, and apigenin have evolved as promising pharmacological agents in the treatment of cancer [37]. It is established that flavonoids and their metabolites, thanks to their both hydrophilic and relatively lipophilic properties, may interact with plasma proteins as well as the polar surface region of phospholipid bilayers in lipoproteins and cell membranes [38]. This protective effect of honey is partly mediated via amelioration of oxidative stress in tissues such as GIT, liver, kidney, pancreas, eye, plasma, red blood cells, and reproductive organs [39–44] which invariably supported our findings.

## 5. Conclusion

The findings presented in this research show that *Acacia* honey may ameliorate oxidative stress in the heart, lung, and kidney of Wistar rats. It also suggests that *Acacia *honey may mitigate the effect of arsenicals-induced oxidative stress implicated in chemical carcinogenesis.

## Figures and Tables

**Table 1:** Redox sensitive biomarkers level in rat heart tissues due to *Acacia* honey and sodium arsenite administration.

Treatment groups (*n* = 5)	MDA (*μ*M)	SOD (U/mL)	Catalase (U/mL)	Glutathione peroxidase (U/mL)	Total protein (mg/mL)
Distilled water (normal control)	1.71 ± 0.12^a^	2.50 ± 0.10^b^	46.33 ± 2.08^c^	51.50 ± 3.94^c^	6.23 ± 0.45^a^
20% honey	1.80 ± 0.14^a^	2.60 ± 0.28^b^	39.00 ± 1.41^b^	50.00 ± 3.61^c^	5.98 ± 0.61^a^
5 mg/kg sodium arsenite	2.95 ± 0.07^b^	1.03 ± 0.00^a^	33.00 ± 0.00^a^	42.00 ± 4.86^a^	6.13 ± 0.19^a^
20% honey + 5 mg/kg sodium arsenite	1.45 ± 0.21^a^	2.25 ± 0.49^b^	40.00 ± 4.24^b^	47.25 ± 2.98^b^	5.85 ± 0.67^a^

MDA: malondialdehyde, SOD: superoxide dismutase. The values are presented as mean ± SD. Values with different superscripts are significantly different from each other at *P* < 0.05 down the column.

**Table 2:** Redox sensitive biomarkers level in rat lung tissues due to *Acacia* honey and sodium arsenite administration.

Treatment groups (*n* = 5)	MDA (*μ*M)	SOD (U/mL)	Catalase (U/mL)	Glutathione peroxidase (U/mL)	Total protein (mg/mL)
Distilled water (normal control)	1.76 ± 0.15^a^	2.30 ± 0.10^c^	54.00 ± 1.00^c^	49.33 ± 3.50^c^	5.88 ± 0.31^a^
20% honey	1.95 ± 0.07^a^	2.05 ± 0.07^b^	50.00 ± 1.41^b^	46.00 ± 2.24^b^	6.08 ± 0.22^a^
5 mg/kg sodium arsenite	2.80 ± 0.14^b^	1.05 ± 0.21^a^	41.00 ± 0.00^a^	41.33 ± 3.44^a^	5.87 ± 0.46^a^
20% honey + 5 mg/kg sodium arsenite	1.70 ± 0.01^a^	1.95 ± 0.07^b^	50.00 ± 4.24^b^	45.00 ± 3.56^b^	6.00 ± 0.14^a^

MDA: malondialdehyde, SOD: superoxide dismutase. The values are presented as mean ± SD. Values with different superscripts are significantly different from each other at *P* < 0.05 down the column.

**Table 3:** Redox sensitive biomarkers level in rat kidney tissues due to *Acacia* honey and sodium arsenite administration.

Treatment groups (*n* = 5)	MDA (*μ*M)	SOD (U/mL)	Catalase (U/mL)	Glutathione peroxidase (U/mL)	Total protein (mg/mL)
Distilled water (normal control)	1.51 ± 0.25^a^	2.30 ± 0.10^b^	61.00 ± 1.00^d^	49.00 ± 3.22^c^	6.20 ± 0.26^a^
20% honey	1.95 ± 0.07^a^	1.90 ± 0.14^ab^	53.50 ± 3.53^c^	43.60 ± 1.52^b^	6.20 ± 0.41^a^
5 mg/kg sodium arsenite	2.75 ± 0.07^b^	1.05 ± 0.07^a^	40.50 ± 0.71^a^	37.17 ± 4.22^a^	5.95 ± 0.27^a^
20% honey + 5 mg/kg sodium arsenite	1.65 ± 0.07^a^	2.10 ± 0.01^b^	47.00 ± 1.41^b^	44.25 ± 1.26^b^	6.05 ± 0.37^a^

MDA: malondialdehyde, SOD: superoxide dismutase. The values are presented as mean ± SD and values with different superscripts are significantly different from each other at *P* < 0.05 down the column.
